# Improvement of Skeletal Fragility by Teriparatide in Adult Osteoporosis Patients: A Novel Mechanostat-Based Hypothesis for Bone Quality

**DOI:** 10.3389/fendo.2015.00006

**Published:** 2015-01-30

**Authors:** Toshihiro Sugiyama, Tetsuya Torio, Tsuyoshi Sato, Masahito Matsumoto, Yoon Taek Kim, Hiromi Oda

**Affiliations:** ^1^Department of Orthopaedic Surgery, Saitama Medical University, Saitama, Japan; ^2^Department of Oral and Maxillofacial Surgery, Saitama Medical University, Saitama, Japan; ^3^Division of Functional Genomics and Systems Medicine, Research Center for Genomic Medicine, Saitama Medical University, Saitama, Japan

**Keywords:** bone, parathyroid hormone, mechanostat, mechanical strain, bone quality

## Skeletal Adaptation to Mechanical Strain in Humans

Several lines of clinical evidence (1–3) suggest that the adult skeleton in humans continuously responds to change in mechanical environment to maintain resultant “elastic” deformation (strain) of bone; increased or decreased bone strain would normally induce bone gain or loss, respectively. Indeed, skeletal adaptation to mechanical strain, known as the mechanostat (4–6), plays a significant role in the treatment of osteoporosis. For example, bone strain from habitual physical activity decreases when an osteoporosis drug increases bone strength, indicating that the effect of osteoporosis therapy is limited by mechanical strain-related feedback control; this mechanostat-based logic is consistent with various clinical data (3). Approaches to reduce the limitation of osteoporosis therapy include pharmacologically enhancing skeletal response to mechanical loading, and earlier experimental studies using external mechanical loading models show that intermittent treatment with parathyroid hormone has such a possibility (7, 8). Importantly, treatment with teriparatide could synergistically produce bone gain with even low, physiological levels of mechanical loading in humans (9) as well as animals (10). The present article concisely discusses the effects of daily or weekly treatment with teriparatide and proposes a new mechanostat-based hypothesis for bone quality associated with mineral versus collagen.

## Daily or Weekly Treatment with Teriparatide in Osteoporosis

In Japan, not only daily subcutaneous injection of teriparatide (20 μg/day) (11–13) but also weekly subcutaneous injection of teriparatide (56.5 μg/week) (14, 15) has been approved for the treatment of adult osteoporosis patients with high risk of fracture. Interestingly, there are marked differences in the effects of these two treatments on circulating markers of bone formation and resorption. The daily injection results in a rapid and sustained increase in bone formation markers followed by a delayed increase in bone resorption markers (12); the period of time during which the increase in bone formation is superior to that in bone resorption is called the anabolic window (16). In contrast, the weekly injection induces only a transient increase in bone formation markers without an increase in bone resorption markers (14).

Formation and resorption occur on different surfaces during bone modeling, and thus modeling-based bone formation and resorption are not coupled; such uncoupling factors include mechanical loading that stimulates bone formation and suppresses bone resorption. Modeling-based bone formation by histomorphometry (17, 18) as well as an increase in bone formation markers and a decrease in bone resorption markers in blood (19) are observed during the first month of daily treatment with teriparatide, which is consistent with clinical finding suggesting that daily treatment with teriparatide and normal physical activity synergistically produce bone gain (9). A rapid but transient increase in bone formation markers without an increase in bone resorption markers (14) implies that weekly treatment with teriparatide also stimulates modeling-based bone formation.

On the other hand, long-term daily, but not weekly, treatment with teriparatide causes increases in both bone formation and resorption markers (12, 14). These systemic changes agree with histomorphometric data showing that 1 or 2 years of daily treatment with teriparatide results in an increase in remodeling-based bone formation (20); resorption followed by formation occurs on the same surface during bone remodeling and thus remodeling-based bone resorption and formation are coupled. Increased or decreased bone remodeling lowers or raises, respectively, the degree of mineralization (21), and cortical volumetric bone mineral density (BMD) is decreased after daily treatment with teriparatide (13). In contrast, weekly treatment with teriparatide is unlikely to increase bone remodeling because neither an increase in bone resorption markers nor a decrease in cortical volumetric BMD is not found (14, 15).

## Perspectives on the Effects of Teriparatide on Bone Fragility

An important goal of osteoporosis therapy is to prevent hip fracture associated with significant morbidity and mortality. The latest systematic review suggests that bone fragility at the hip is improved by daily treatment with teriparatide (22); the effect of weekly treatment with teriparatide on non-vertebral fracture risk is under investigation. Here, we present mechanostat-based perspectives on this topic.

**Figure 1:**
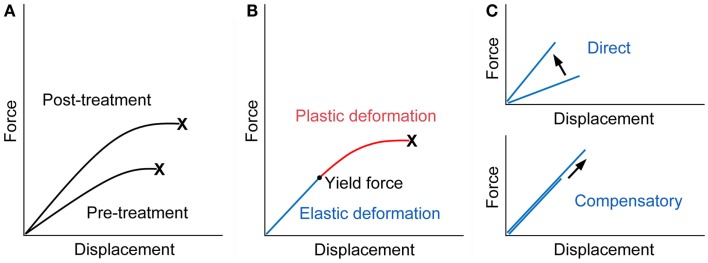
**Force displacement curve of a bone**. **(A)** Treatment with an ideal osteoporosis drug improves bone fragility by increasing both of the force and displacement at failure (23). X denotes fracture. **(B)** The curve would consist of the pre-yield “elastic” deformation associated with mineral and the post-yield “plastic” deformation associated with collagen. Consequently, mechanical strain-related feedback control, the mechanostat, could work against mineral-related, but not collagen-related, impairment of bone quality. X denotes fracture. **(C)** The pre-yield “elastic” deformation can be modified by osteoporosis therapy that (i) directly enhances the response to mechanical loading and increases the slope of the curve (upper) or (ii) lowers mineral-related bone quality and results in compensatory bone gain by the mechanostat to maintain the slope of the curve (lower). Note that, in the latter case, the yield force can be increased if compensated efficiently.

Fall-related fracture occurs if the energy from the fall is higher than that the bone can absorb. Force displacement curve obtained from a biomechanical test, in which a bone is loaded until it fractures, shows that work to failure (energy absorption), the area under the curve, represents bone fragility, and an ideal strategy for the improvement of bone fragility is to increase both the force and displacement at failure (23) (Figure 1A).

From a material point of view, stiffness and toughness of bone tissue generally depend on mineral and collagen, respectively (24). There is a yield force at which a bone begins to deform plastically, and mechanical strain from normal physical activity would be linked to the pre-yield “elastic” deformation associated with mineral but not to the post-yield “plastic” deformation associated with collagen (Figure 1B). Consequently, mechanical strain-related feedback control could compensate mineral-related, but not collagen-related, impairment of bone quality to maintain “elastic” deformation. Indeed, this theory is compatible with clinical data relating to bone quality. Examples of the mechanostat-based compensation for mineral-related impairment of bone quality would include rickets/osteomalacia and use of warfarin (3, 25–27), while the impairment of bone quality associated with collagen cross-links significantly contributes to skeletal fragility in diabetes (28–30).

It is possible to speculate that treatment with teriparatide improves bone fragility at the hip through the mechanostat-based “modeling-related direct” and “remodeling-related compensatory” mechanisms (Figure 1C). Both daily and weekly treatments are expected to have the former effect by the enhancement of skeletal response to mechanical loading (7–10). In contrast, the latter effect is linked to daily treatment; a decrease in the degree of mineralization after daily but not weekly treatment (13, 15) might act to improve bone fragility if compensated efficiently, because compensatory bone gain by the mechanostat to maintain the pre-yield “elastic” deformation could increase the yield force at which a bone begins to deform plastically and thus the energy that the bone can absorb. This possibility is supported by histomorphometric data showing that one or two years of the treatment results in increases in modeling- and remodeling-based bone formation (20), because the mechanosat suggests that the former “modeling-related direct” effect does not continue for a long time (3).

Finally, the mechanostat-based theory appears to be inconsistent with clinical data that daily or weekly treatment with teriparatide stimulates bone accrual at the endosteal rather than periosteal surface, because the strain level would be lower at the former site; endosteal as well as trabecular, but not periosteal, bone apposition is detected by computed tomography after daily (13) and weekly (15) treatments. Teriparatide-induced bone modeling is dose-dependent (17, 18), implying higher concentrations of the agent at the trabecular and endosteal surfaces. Regardless of the mechanism, the mechanostat suggests that inner bone gain could limit outer bone gain, because bone gain in the inner compartments is likely to decrease bone strain in the outer compartment.

## Conflict of Interest Statement

The authors declare that the research was conducted in the absence of any commercial or financial relationships that could be construed as a potential conflict of interest.

## References

[1] Christen P Ito K Ellouz R Boutroy S Sornay-Rendu E Chapurlat RD Bone remodelling in humans is load-driven but not lazy. Nat Commun (2014) 5:4855. 10.1038/ncomms5855 25209333

[2] Bhatia VA Edwards WB Johnson JE Troy KL. Short-term bone formation is greatest within high strain regions of the human distal radius: a prospective pilot study. J Biomech Eng (2015) 137:011001. 10.1115/1.4028847 25322335 PMC4296241

[3] Sugiyama T Kim YT Oda H. Osteoporosis therapy: a novel insight from natural homeostatic system in the skeleton. Osteoporos Int (2015). 10.1007/s00198-014-2923-y 25288445

[4] Frost HM. Bone’s mechanostat: a 2003 update. Anat Rec A Discov Mol Cell Evol Biol (2003) 275:1081–101. 10.1002/ar.a.10119 14613308

[5] Skerry TM. The response of bone to mechanical loading and disuse: fundamental principles and influences on osteoblasts/osteocyte homeostasis. Arch Biochem Biophys (2008) 473:117–23. 10.1016/j.abb.2008.02.028 18334226

[6] Meakin LB Price JS Lanyon LE. The contribution of experimental in vivo models to understanding the mechanisms of adaptation to mechanical loading in bone. Front Endocrinol (2014) 5:154. 10.3389/fendo.2014.00154 25324829 PMC4181237

[7] Chow JW Fox S Jagger CJ Chambers TJ. Role for parathyroid hormone in mechanical responsiveness of rat bone. Am J Physiol (1998) 274:E146–54. 9458760 10.1152/ajpendo.1998.274.1.E146

[8] Hagino H Okano T Akhter MP Enokida M Teshima R. Effect of parathyroid hormone on cortical bone response to in vivo external loading of the rat tibia. J Bone Miner Metab (2001) 19:244–50. 10.1007/s007740170027 11448017

[9] Poole KE Treece GM Ridgway GR Mayhew PM Borggrefe J Gee AH. Targeted regeneration of bone in the osteoporotic human femur. PLoS One (2011) 6:e16190. 10.1371/journal.pone.0016190 21264263 PMC3021547

[10] Sugiyama T Saxon LK Zaman G Moustafa A Sunters A Price JS Mechanical loading enhances the anabolic effects of intermittent parathyroid hormone (1-34) on trabecular and cortical bone in mice. Bone (2008) 43:238–48. 10.1016/j.bone.2008.04.012 18539556

[11] Neer RM Arnaud CD Zanchetta JR Prince R Gaich GA Reginster JY Effect of parathyroid hormone (1-34) on fractures and bone mineral density in postmenopausal women with osteoporosis. N Engl J Med (2001) 344:1434–41 10.1056/NEJM200105103441904 11346808

[12] Miyauchi A Matsumoto T Sugimoto T Tsujimoto M Warner MR Nakamura T. Effects of teriparatide on bone mineral density and bone turnover markers in Japanese subjects with osteoporosis at high risk of fracture in a 24-month clinical study: 12-month, randomized, placebo-controlled, double-blind and 12-month open-label phases. Bone (2010) 47:493–502. 10.1016/j.bone.2010.05.022 20580870

[13] Borggrefe J Graeff C Nickelsen TN Marin F Gluer CC. Quantitative computed tomographic assessment of the effects of 24 months of teriparatide treatment on 3D femoral neck bone distribution, geometry, and bone strength: results from the EUROFORS study. J Bone Miner Res (2010) 25:472–81. 10.1359/jbmr.090820 19778182

[14] Nakamura T Sugimoto T Nakano T Kishimoto H Ito M Fukunaga M Randomized Teriparatide [human parathyroid hormone (PTH) 1-34] once-weekly efficacy research (TOWER) trial for examining the reduction in new vertebral fractures in subjects with primary osteoporosis and high fracture risk. J Clin Endocrinol Metab (2012) 97:3097–106 10.1210/jc.2011-3479 22723322

[15] Ito M Oishi R Fukunaga M Sone T Sugimoto T Shiraki M The effects of once-weekly teriparatide on hip structure and biomechanical properties assessed by CT. Osteoporos Int (2014) 25:1163–72. 10.1007/s00198-013-2596-y 24345886 PMC3923120

[16] Rubin MR Bilezikian JP The anabolic effects of parathyroid hormone therapy. Clin Geriatr Med (2003) 19:415–32 10.1016/S0749-0690(02)00074-5 12916294

[17] Lindsay R Cosman F Zhou H Bostrom MP Shen VW Cruz JD A novel tetracycline labeling schedule for longitudinal evaluation of the short-term effects of anabolic therapy with a single iliac crest bone biopsy: early actions of teriparatide. J Bone Miner Res (2006) 21:366–73. 10.1359/JBMR.051109 16491283

[18] Lindsay R Zhou H Cosman F Nieves J Dempster DW Hodsman AB. Effects of a one-month treatment with PTH(1-34) on bone formation on cancellous, endocortical, and periosteal surfaces of the human ilium. J Bone Miner Res (2007) 22:495–502. 10.1359/jbmr.070104 17227219

[19] Glover SJ Eastell R McCloskey EV Rogers A Garnero P Lowery J Rapid and robust response of biochemical markers of bone formation to teriparatide therapy. Bone (2009) 45:1053–8. 10.1016/j.bone.2009.07.091 19679211

[20] Ma YL Zeng Q Donley DW Ste-Marie LG Gallagher JC Dalsky GP Teriparatide increases bone formation in modeling and remodeling osteons and enhances IGF-II immunoreactivity in postmenopausal women with osteoporosis. J Bone Miner Res (2006) 21:855–64. 10.1359/jbmr.060314 16753016

[21] Roschger P Misof B Paschalis E Fratzl P Klaushofer K. Changes in the degree of mineralization with osteoporosis and its treatment. Curr Osteoporos Rep (2014) 12:338–50. 10.1007/s11914-014-0218-z 24947951

[22] Eriksen EF Keaveny TM Gallagher ER Krege JH. Literature review: the effects of teriparatide therapy at the hip in patients with osteoporosis. Bone (2014) 67:246–56. 10.1016/j.bone.2014.07.014 25053463

[23] Turner CH. Biomechanics of bone: determinants of skeletal fragility and bone quality. Osteoporos Int (2002) 13:97–104. 10.1007/s001980200000 11905527

[24] Fratzl P Gupta HS Paschalis EP Roschger P Structure and mechanical quality of the collagen-mineral nano-composite in bone. J Mater Chem (2004) 14:2115–23 10.1039/b402005g

[25] Sugiyama T Tanaka S Miyajima T Kim YT Oda H Vitamin D supplementation and fracture risk in adults: a new insight. Osteoporos Int (2014) 25:2497–8 10.1007/s00198-014-2798-y 24989078

[26] Sugiyama T Yoshioka H Sakaguchi K Kim YT Oda H An evidence-based perspective on vitamin D and the growing skeleton. Osteoporos Int (2015). 10.1007/s00198-014-2975-z 25448838

[27] Sugiyama T Kugimiya F Kono S Kim YT Oda H Warfarin use and fracture risk: an evidence-based mechanistic insight. Osteoporos Int (2015). 10.1007/s00198-014-2912-1 25300528

[28] Leslie WD Rubin MR Schwartz AV Kanis JA. Type 2 diabetes and bone. J Bone Miner Res (2012) 27:2231–7. 10.1002/jbmr.1759 23023946

[29] Garnero P. The contribution of collagen crosslinks to bone strength. Bonekey Rep (2012) 1:182. 10.1038/bonekey.2012.182 24363926 PMC3868729

[30] Saito M Marumo K Bone quality in diabetes. Front Endocrinol (2013) 4:72 10.3389/fendo.2013.00072 PMC3682213 23785354

